# Use of clinical decision support for antibiotic stewardship in the emergency department and outpatient clinics: An interrupted time-series analysis

**DOI:** 10.1017/ash.2023.140

**Published:** 2023-04-26

**Authors:** James S. Ford, Brittany L. Morgan Bustamante, Mehr Kaur Virk, Nancy Ramirez, Cynthia G. Matsumoto, Daniel Jin Lee, Scott MacDonald, Larissa May

**Affiliations:** 1 Department of Emergency Medicine, University of California–San Francisco, San Francisco, California; 2 Department of Public Health Sciences, University of California–Davis School of Medicine, Davis, California; 3 Department of Emergency Medicine, University of California Davis, School of Medicine. Sacramento, California; 4 Office of Population Health and Accountable Care, University of California Davis, Sacramento, California; 5 Department of Clinical Informatics, University of California Davis Health, Sacramento, California

## Abstract

**Objective:**

To evaluate the impact of implementing clinical decision support (CDS) tools for outpatient antibiotic prescribing in the emergency department (ED) and clinic settings.

**Design:**

We performed a before-and-after, quasi-experimental study that employed an interrupted time-series analysis.

**Setting:**

The study institution was a quaternary, academic referral center in Northern California.

**Participants:**

We included prescriptions for patients in the ED and 21 primary-care clinics within the same health system.

**Intervention:**

We implemented a CDS tool for azithromycin on March 1, 2020, and a CDS tool for fluoroquinolones (FQs; ie, ciprofloxacin, levofloxacin, and moxifloxacin) on November 1, 2020. The CDS added friction to inappropriate ordering workflows while adding health information technology (HIT) features to easily perform recommended actions. The primary outcome was the number of monthly prescriptions for each antibiotic type, by implementation period (before vs after).

**Results:**

Immediately after azithromycin-CDS implementation, monthly rates of azithromycin prescribing decreased significantly in both the ED (−24%; 95% CI, −37% to −10%; *P* < .001) and outpatient clinics (−47%; 95% CI, −56% to −37%; *P* < .001). In the first month after FQ-CDS implementation in the clinics, there was no significant drop in ciprofloxacin prescriptions; however, there was a significant decrease in ciprofloxacin prescriptions over time (−5% per month; 95% CI, −6% to −3%; *P* < .001), suggesting a delayed effect of the CDS.

**Conclusion:**

Implementing CDS tools was associated with an immediate decrease in azithromycin prescriptions, in both the ED and clinics. CDS may serve as a valuable adjunct to existing antimicrobial stewardship programs.

The World Health Organization (WHO) declared antimicrobial resistance (AMR) as one of the top 10 global public health threats facing humanity.^1^ In 2019, there were nearly 5 million global deaths associated with AMR, and this number is expected to double by 2050.^2,3^ Inappropriate antibiotic use has been identified as the most important preventable cause of drug resistance in both inpatient and outpatient settings.^4,5^ Outpatient encounters, which include emergency department (ED) and clinic visits, account for 80%–90% of antimicrobial prescriptions worldwide.^6^ Despite a growing movement to improve antibiotic prescription practices, inappropriate antibiotic use in EDs and clinics remains widespread.^7^ In the United States, suboptimal antibiotic use has been identified 33%–57% of ED prescriptions and 30%–82% of clinic prescriptions.^8–11^

Antimicrobial stewardship programs have historically targeted inpatient antimicrobial use. Previous studies in the inpatient setting have reported that antimicrobial stewardship can reduce inappropriate antibiotic use, lower costs, decrease treatment duration, decrease adverse effects related to antimicrobial use, and reduce local AMR.^12,13^ Recently, increased interest has been placed on outpatient antimicrobial stewardship programs, and in 2020, the US government released the National Plan for Combating Antibiotic-Resistant Bacteria, which called for a reduction of outpatient antibiotic prescription use.^14^ New studies in the outpatient setting have shown that antimicrobial stewardship interventions can reduce inappropriate antibiotic prescribing.^15^ However, few high-quality, ED-based studies of antimicrobial stewardship programs have been published.^16,17^

The use of digital resources such as computerized clinical decision support (CDS) can play an important role in promoting antimicrobial stewardship. CDS is a set of robust clinical decision-making tools that provide evidence-based clinical recommendations that can be implemented by providers, and these tools have proven useful in the outpatient clinic setting.^15^ However, few if any studies have assessed the utility of ED-based, antimicrobial stewardship programs that employ CDS systems. In this study, we evaluated the impact of implementing an intervention for antimicrobial stewardship that employs an electronic health record (EHR)–based CDS, in the ED and clinic settings.

## Methods

### Brief overview of antimicrobial stewardship program and CDS tools

In 2017, the study institution implemented a comprehensive, ED-based, antibiotic stewardship program that focused on reducing inappropriate antibiotic use for respiratory tract infections and skin and soft-tissue infections. In 2018, the ED antibiotic stewardship program expanded to clinics within the same health network. We used implementation science methodology to conduct in-depth interviews and provider surveys to identify areas of improvement, and we used commitment logs and public commitment posters to encourage provider buy-in. These programs have been previously described.^16,18^

To improve the institutional antibiotic stewardship program, 2 CDS tools were implemented that were programmed to trigger when azithromycin or fluoroquinolones (FQs; ie, ciprofloxacin, levofloxacin, or moxifloxacin) were ordered by providers in the ED and outpatient clinics. These antibiotics were targeted because they were identified to be overused, particularly for respiratory tract infections, based on our internal outpatient AMS program data. Using principles of sound CDS design, our approach was to add friction to inappropriate ordering workflows, while adding health information technology (HIT) features to easily perform recommended actions.^19^ Users could choose an order with a preset indication from a list, or by searching for the indication itself. If ordering the antibiotic without indication, the system required the selection of an indication from a preset list of appropriate indications. For indications not listed, the option of “other” indication required the indication to be manually typed out. The CDS tool for azithromycin (Supplementary Figs. 1 and 2) was implemented on March 1, 2020, and the CDS tool for FQs was implemented on November 1, 2020. The CDS was suppressed for antibiotic reorders. After implementation, it came to our attention that the CDS had been suppressed for pre-existing personal preference lists. Clinicians received education on how to use these CDS tools via email. The CDS was implemented into our EHR, Epic (Epic Systems, Verona, WI).

**Fig. 1.**
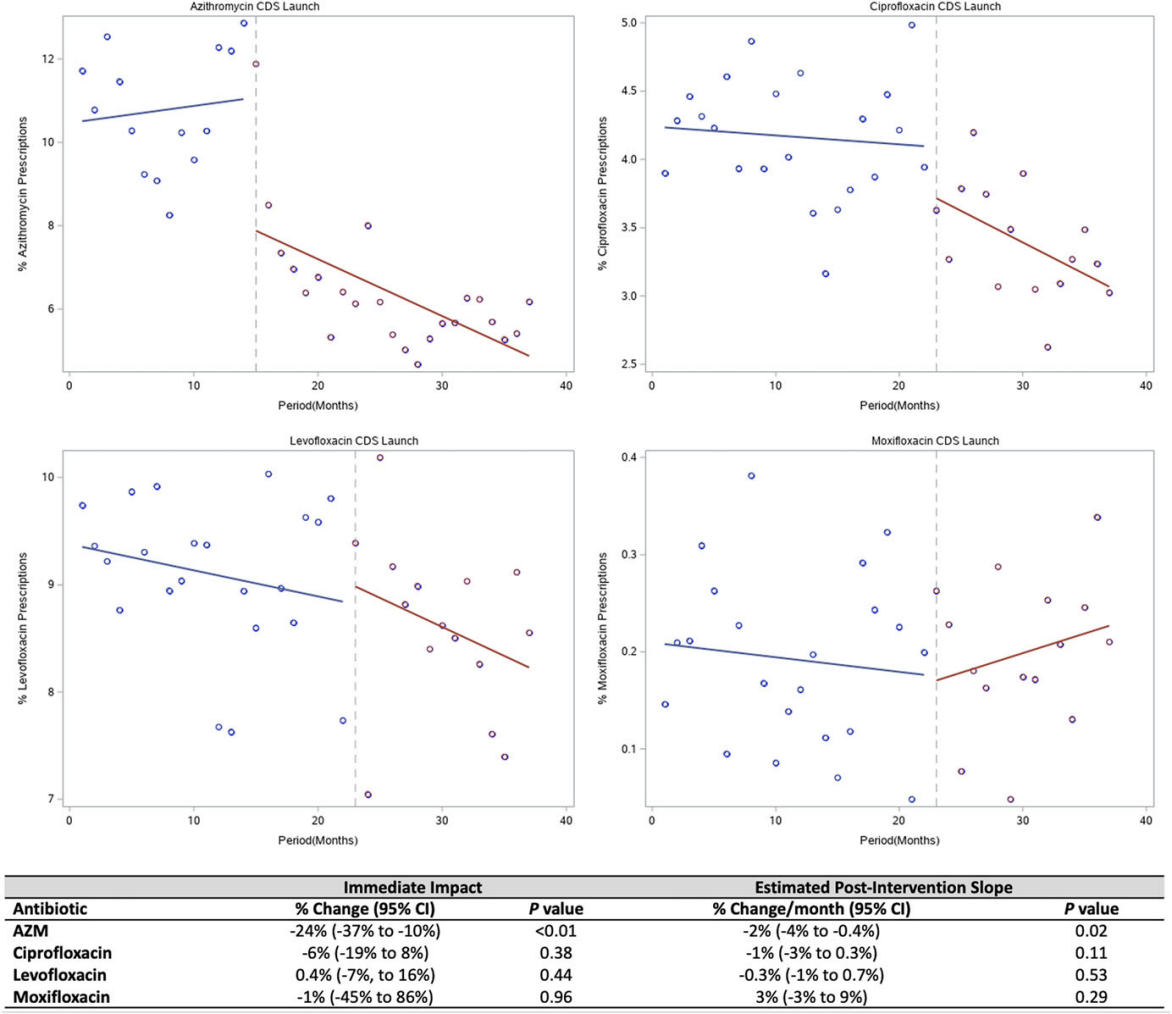
Interrupted time-series analysis of antibiotic prescriptions in the emergency department. Azithromycin and fluoroquinolone prescriptions are displayed as a proportion of total prescriptions in the emergency department.

**Fig. 2.**
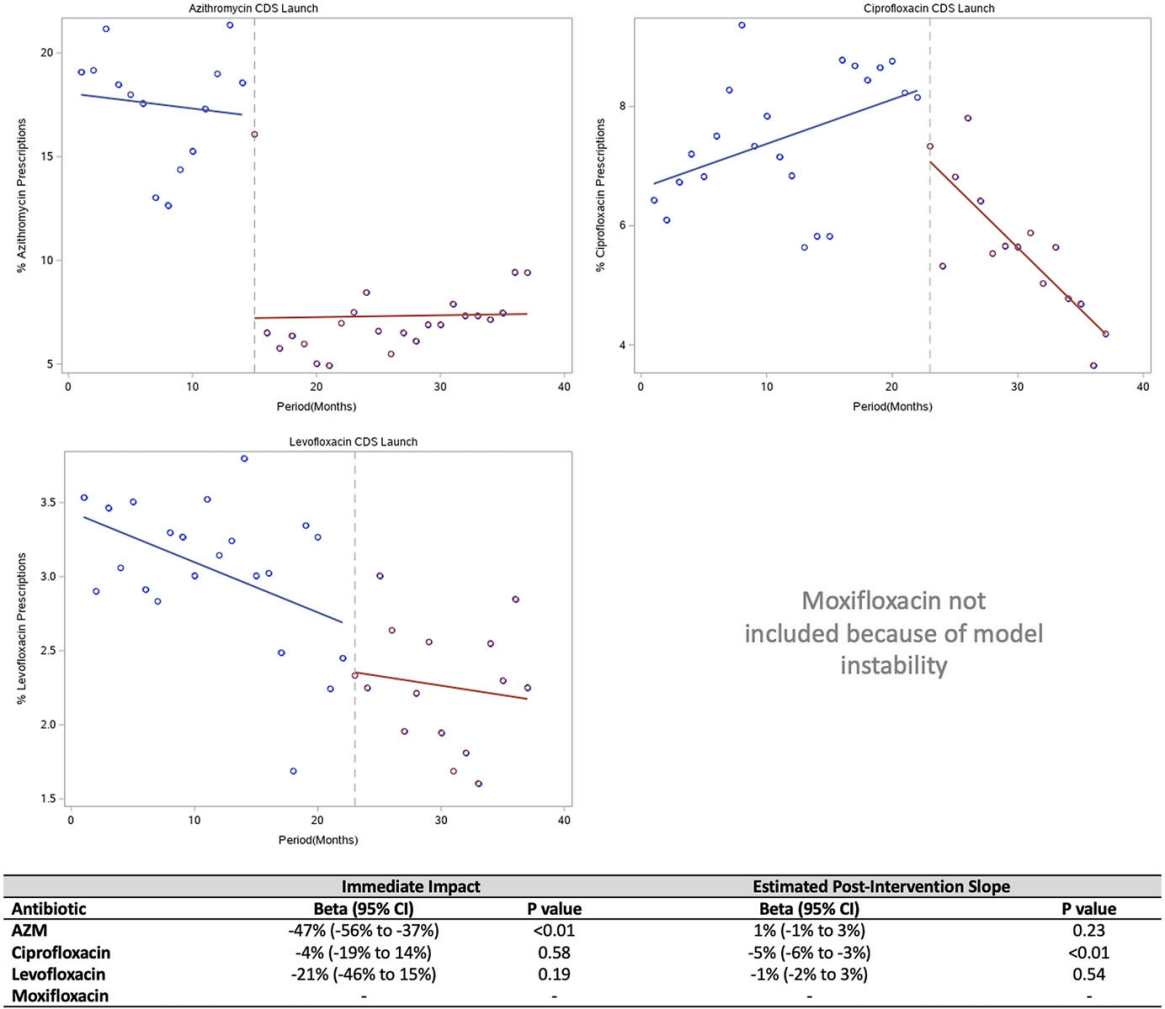
Interrupted time-series analysis of antibiotic prescriptions in the outpatient clinics. Azithromycin and fluoroquinolone prescriptions are displayed as a proportion of total prescriptions in the clinics.

### Study setting

The study institution was a quaternary care, academic referral center in Northern California. The study ED triages >85,000 patients and clinic network services 1.1 million patient visits per year. The system provides care for a mixed urban and rural population. The study institution has a regional network of outpatient clinics that includes 31 primary-care clinics in Sacramento and surrounding cities.

### Study design

We used a before-and-after, quasi-experimental design to assess the effect of the implementation of our CDS tools.^20^ For azithromycin, the preimplementation period included prescriptions that were made between January 1, 2019, and February 28, 2020 (14 months) and the postimplementation period included prescriptions that were made between March 1, 2020, and January 31, 2022 (22 months). For FQ prescriptions, the preimplementation period included all prescriptions that were made between January 1, 2019, and October 31, 2020 (22 months), and the postimplementation period included all prescriptions made between November 1, 2020, and January 31, 2022 (14 months). Data were abstracted through EMR computer-generated reports and included information on CDS triggering, clinician response, prescription date, and prescription type. The primary outcome was the number of monthly prescriptions for each antibiotic type. This study was approved under exempt status by the study site’s institutional review board quality improvement self-certification tool.

### Statistical analysis

To assess the impact of CDS implementation over time, we performed an interrupted time-series (ITS) analysis that adjusted for seasonality, pre-CDS prescribing trends, and temporal autocorrelation. The number of CDS-eligible antibiotic prescriptions (azithromycin and FQs) and the total number of all antibiotic prescriptions were aggregated at the month level, resulting in 37 data points. The outcome variable was the number of CDS-eligible prescriptions. A sequential period variable (months from the start of the study period), a binary intervention variable, a continuous time after the intervention variable (number of months elapsed since CDS implementation), and a seasonality variable to account for flu season from October through April (as defined by the United States Centers for Disease Control and Prevention) were included in the model.^21^

A segmented regression analysis was performed using a negative binomial, mixed-effects model of the monthly data (ie, the Glimmix procedure). Separate models were built for each antibiotic (azithromycin, ciprofloxacin, levofloxacin, and moxifloxacin). The effect of CDS implementation was assessed separately for the ED and clinics. Period, intervention, and time after intervention were included as fixed effects (ie, study-wide effects). A random residual with an autoregressive variance structure was used to account for the correlation of repeated measures taken over time, across clinics, and overdispersion. To account for the differing effects of CDS implementation by clinic, a random intercept and intervention terms were included in the clinic model. To help compare rates across periods, the total number of prescriptions written (at each location per month) was used as an offset variable, acting as the denominator for the primary outcome of interest. All statistical tests were 2-sided, and *P* values < .05 were considered significant. Statistical analyses were performed using SAS Studio software (SAS Institute, Cary, NC).

## Results

### Overall antibiotic prescription trends

Of the 4 antibiotics for which we measured prescription use, azithromycin and levofloxacin were the most prescribed in the ED, and azithromycin and ciprofloxacin were the most prescribed in the clinics. In the ED, all antibiotic prescriptions fell from 4,486 per month to 4,184 per month after implementation of the azithromycin CDS tool, and these rates remained stable after FQ CDS implementation: 4,305 before the intervention versus 4,308 after the intervention. In the clinics, all antibiotic prescriptions fell from 3,354 per month to 2,177 per month after implementation of the azithromycin CDS tool, and all antibiotic prescriptions fell from 2,777 per month to 2,172 per month after FQ CDS implementation. Total prescriptions per month, by drug, are described in Table 1. The most common indications for each antibiotic, by clinical setting, are described in Table 2.

**Table 1.** Antibiotic Prescriptions Per Month by Study Period and Practice Setting

	Before CDS		After CDS	
Antibiotic	Mean No./Month (±SD)	%	Mean No./Month (±SD)	%
**Emergency department**				
Azithromycin	485 (±81)	11	265 (±60)	6
All antibiotics^a^	4,486 (±246)	…	4,184 (±352)	…
Ciprofloxacin	179 (±23)	4	146 (±16)	3
Levofloxacin	391 (±40)	9	370 (±37)	9
Moxifloxacin	8 (±4)	<1	9 (±1)	9
All antibiotics^b^	4,305 (±365)	…	4,308 (±323)	…
**Clinics**				
Azithromycin	46 (±32)	0–30	13 (±12)	0–22
All antibiotics^a^	259 (±143)	...	174 (±90)	...
Ciprofloxacin	17 (±9)	0–30	9 (±6)	0–17
Levofloxacin	7 (±5)	0–12	4 (±3)	0–13
Moxifloxacin	<1 (±1)	0–4	<1 (±1)	0–7
All antibiotics^b^	231 (±135)	…	169 (±84)	…

Note. CDS, clinical decision support intervention.

a Represents total antibiotics prescribed during the study periods before and after implementation of the azithromycin CDS.

b Represents total antibiotics prescribed during the study periods before and after implementation of the FQ CDS.

**Table 2.** Antibiotic Prescription Indications by Study Setting

Antibiotic	Top 3 Indications	
	Emergency Department	Clinics
Azithromycin	1. COPD exacerbation, 36%	1. Other, 41%
	2. CAP, 38%	2. Bacterial sinusitis, 13%
	3. Other, 18%	3. COPD exacerbation, 13%
Ciprofloxacin	1. Other, 36%	1. Complicated UTI, 37%
	2. Diverticulitis, 24%	2. Other, 28%
	3. Complicated UTI, 19%	3. Diverticulitis, 23%
Levofloxacin	1. Other, 38%	1. Other, 31%
	2. Complicated UTI, 21%	2. Complicated UTI, 25%
	3. Pyelonephritis, 18%	3. CAP, 17%
Moxifloxacin	1. Other, 93%	1. Other, 84%
	2. CAP, 7%	2. CAP, 16%
	3. N/A (no other indications listed)	3. N/A

Note. COPD, chronic obstructive pulmonary disease; CAP, community-associated pneumonia; UTI, urinary tract infection; N/A, not available.

### Interrupted time-series analysis

In the ED, the rate of azithromycin prescribing decreased by 24% (95% CI, −38% to −10%; *P* < .001) in the period immediately following CDS implementation. Additionally, azithromycin prescribing continued to decrease throughout the postimplementation period (−2% per month, 95% CI, −4% to −0.4%; *P* = .02). The overall rate of azithromycin prescription declined by 45% between the pre- and the postimplementation periods: 485 per month before the intervention versus 265 per month after the intervention (*P* < .0001). The rate of CDS-eligible prescriptions for FQs did not change significantly immediately following CDS implementation. Figure 1 displays azithromycin and FQ prescriptions as a percentage of all prescriptions in the ED over time.

In the clinic, azithromycin prescriptions decreased by 47% (95% CI, −56% to −37%; *P* < .001). This decrease remained stable over time (1%; 95% CI, −1% to 3%; *P* = .23). The rate of ciprofloxacin prescriptions did not significantly decrease immediately following CDS implementation (−4%; 95% CI, −19% to 14%; *P* = .58). However, there was a significant decrease in ciprofloxacin prescribing over time (−5%; 95% CI, −6% to −3%; *P* < .001). Levofloxacin prescriptions did not decrease significantly following CDS implementation. Too few moxifloxacin prescriptions were written in the clinics to develop a stable model. Figure 2 displays azithromycin and FQ prescriptions as a percentage of all prescriptions in the clinics over time.

### Sensitivity analysis

After controlling for seasonality, there was a significant decrease in the total number of monthly ED antibiotic prescriptions immediately after the azithromycin CDS launch (−17%; 95% CI, −26% to −8%; *P* = .002), and this effect was sustained over time (0.9%; 95% CI, −0.3% to 2%; *P* = .12). The rate of penicillin and cephalosporin prescriptions did not change immediately after the CDS launch. Still, we observed a statistically significant increase over time in those prescriptions after FQ-CDS implementation (1%; 95% CI, 0.3% to 2%; *P* = .01) (Supplementary Fig. 3).

In the clinics, there was a statistically significant decrease in all antibiotic prescriptions after CDS implementation (after azithromycin CDS, −15%; 95% CI, −23% to −7%; *P* = .003; after FQ CDS, −14%; 95% CI, −23% to −5%; *P* = .009). There were statistically significant decreases in penicillin and cephalosporin prescribing immediately following CDS implementation (after azithromycin CDS, −18%; 95% CI, −30% to −5%; *P* = .01; after FQ CDS, −17%; 95% CI, −28% to −4%; *P* = .01). However, there was a significant increase in these prescriptions over time (after azithromycin CDS, 2%; 95% CI, 0.6% to 3%; *P* = .004; after FQ CDS, 5%; 95% CI, 4% to 6%; *P* < .0001).

## Discussion

Given the continued emergence of multidrug-resistant organisms and the increasing complexity of modern healthcare, new strategies are needed to help preserve the integrity of our existing arsenal of antimicrobials. To our knowledge, this study is the first to evaluate the impact of CDS tools for prescribing azithromycin and FQs in the ED and in the outpatient clinics of a single health system.

Previously published literature has had mixed results concerning the efficacy of CDS for antimicrobial stewardship. In general, where broad-spectrum antibiotic (BSA) use was already low, CDS tools did little to affect change.^22^ However, where BSA use was common, CDS tools led to a decrease in inappropriate prescriptions in both the clinic and ED settings.^19,23^

In our study, immediately after azithromycin-CDS implementation, rates of azithromycin prescribing decreased significantly in both the ED and outpatient clinics. Over the course of the postimplementation period, the effect of the CDS on lowering azithromycin prescribing was stable in clinics and continued to decrease in the ED.

By coincidence, the implementation date of the azithromycin CDS tool corresponded approximately to the emergence of the COVID-19 pandemic (March 2020).^24^ Because of this, it was impossible to separate the impact of the pandemic versus the impact of the azithromycin CDS tool. Previous studies in the urgent care and primary care settings found that outpatient antibiotic prescriptions decreased following the start of the COVID-19 pandemic.^25,26^ In our study, overall outpatient prescriptions decreased after March 2020, which suggests that the COVID-19 pandemic may have been at least partially responsible for this drop. However, several months following March 2020 (implementation of azithromycin CDS and start of COVID-19 pandemic), cephalosporin and penicillin use increased as a proportion of all antibiotics prescribed in both the clinics and ED. This trend continued even after the implementation of the FQ CDS. Our study data suggest that there was a shift in prescribing behavior away from azithromycin and towards cephalosporins and penicillin.

The CDS tool for azithromycin was more successful than the CDS tool for FQs. As mentioned, the COVID-19 pandemic may have confounded this result. Alternatively, we suspect that this result may have been influenced by the indication for use and availability of suitable alternatives. For example, “COPD exacerbation” was the most common indication for azithromycin use in the ED and the third most common indication in the clinics. Although there is no clear consensus regarding which antibiotic is best suited for treating COPD exacerbations, clinical practice is highly variable and there are many options other than azithromycin, including doxycycline, trimethoprim-sulfamethoxazole, and cephalosporins, among others.^27^ The second most common indication for azithromycin prescription in the ED was for community-acquired pneumonia (CAP). In October 2019, the Infectious Disease Society of America and the American Thoracic Society released clinical practice guidelines that recommended use of azithromycin for CAP only where local *S. pneumoniae* resistance was <25%.^28^ Although our institution does not track *S. pneumoniae* resistance, we know that resistance in most large institutions high (>25%).^29^ Since we measured the effect of the CDS during the postimplementation period (March 1, 2020, to January 31, 2022), it is possible that concurrent publishing of these guidelines may have affected azithromycin prescription use at the tail end of the postimplementation period for the azithromycin CDS. Nonetheless, the availability of suitable alternatives to azithromycin for treating CAP likely allowed for a decline in azithromycin prescriptions. Conversely, one of the most common indications for FQ use in the ED and the clinics was drug-resistant UTI or complicated UTI.^30^ There are notably fewer antibiotic choices for treating complicated UTI or pyelonephritis, compared to other outpatient infections. Although data regarding which suspected bacterial species were not available, few oral alternatives to FQs for treating suspected or confirmed pseudomonal infections are widely available, making it difficult to affect the prescribing behavior of these drugs.

Our CDS approach was different from conventional CDS systems that employ interruptive alerts. Interruptive alerts are prone to alert fatigue, which leads clinicians to disregard them, often without considering or even reading the CDS content.^31,32^ In our configuration, ordering from a list or searching for an accepted indication did not add clicks to the workflow, and ordering for an appropriate indication only required 1 click. Inappropriate ordering added 1 click and additional typing. We hypothesize that the improvement over time was driven by prescribers learning the easiest, lowest-friction workflow, which is congruent with antimicrobial stewardship standards.

This study had several limitations, including those inherent to its quasi-experimental design. Although a randomized control trial would have been preferable, it would have been difficult within a single health system, given the impracticalities of clustering an intervention of this type at the individual level. The preimplementation and postimplementation study periods were not standardized by length. However, since we used averages at the month level, this should not have influenced our findings. Furthermore, we employed an ITS analysis that attempted to control for secular trends, such as seasonality, and total prescriptions written, by including these as variables in our mixed-effects, negative binomial model. Due to a small sample size, we were unable to perform an ITS analysis for moxifloxacin prescriptions in the clinic setting, so we cannot comment on the effect of the intervention on the prescription of this drug. We did not assess appropriateness of prescription use, we can only comment on global trends in antibiotic use. After CDS implementation, it came to our attention that the CDS had been suppressed for pre-existing personal preference lists, which may have limited its effect. Finally, this study was performed within a single health system, which limits its generalizability.

In conclusion, using CDS was associated with an immediate and sustained decrease in azithromycin prescriptions in the ED and an immediate decrease in azithromycin prescriptions in the clinics. The CDS was also associated with a delayed decrease in ciprofloxacin prescriptions in the clinics. CDS may serve as a valuable adjunct to existing antimicrobial stewardship programs.

## Supplementary material

For supplementary material accompanying this paper visit http://doi.org/10.1017/ash.2023.140. click here to view supplementary material
