# Supplementary figures and images for: Use of clinical decision support for antibiotic stewardship in the emergency department and outpatient clinics: An interrupted time-series analysis
Source: Antimicrob Steward Healthc Epidemiol. 2023 Apr 26;3(1):e80. doi: 10.1017/ash.2023.140 (PMC10186615; doi:10.1017/ash.2023.140)

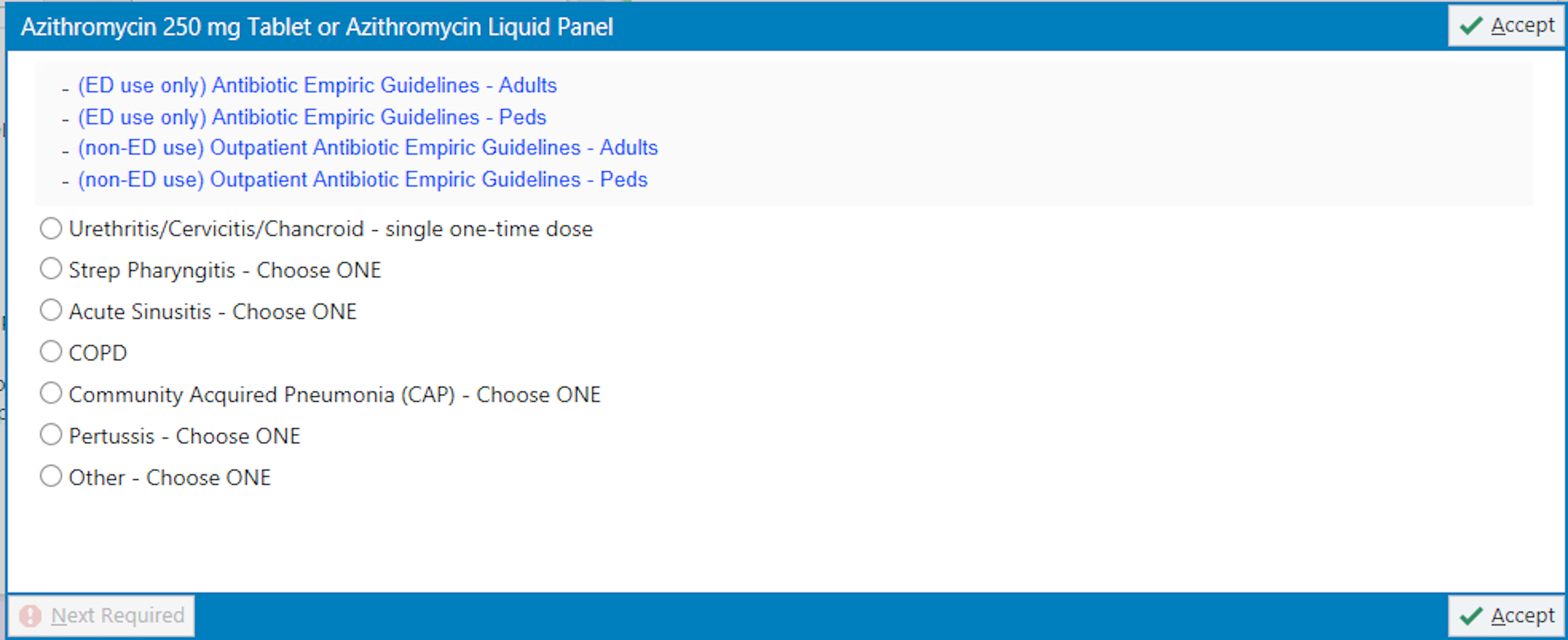

Supplement: Supplementary file 1 [file ashsup.zip › S2732494X23001407sup001.jpg]

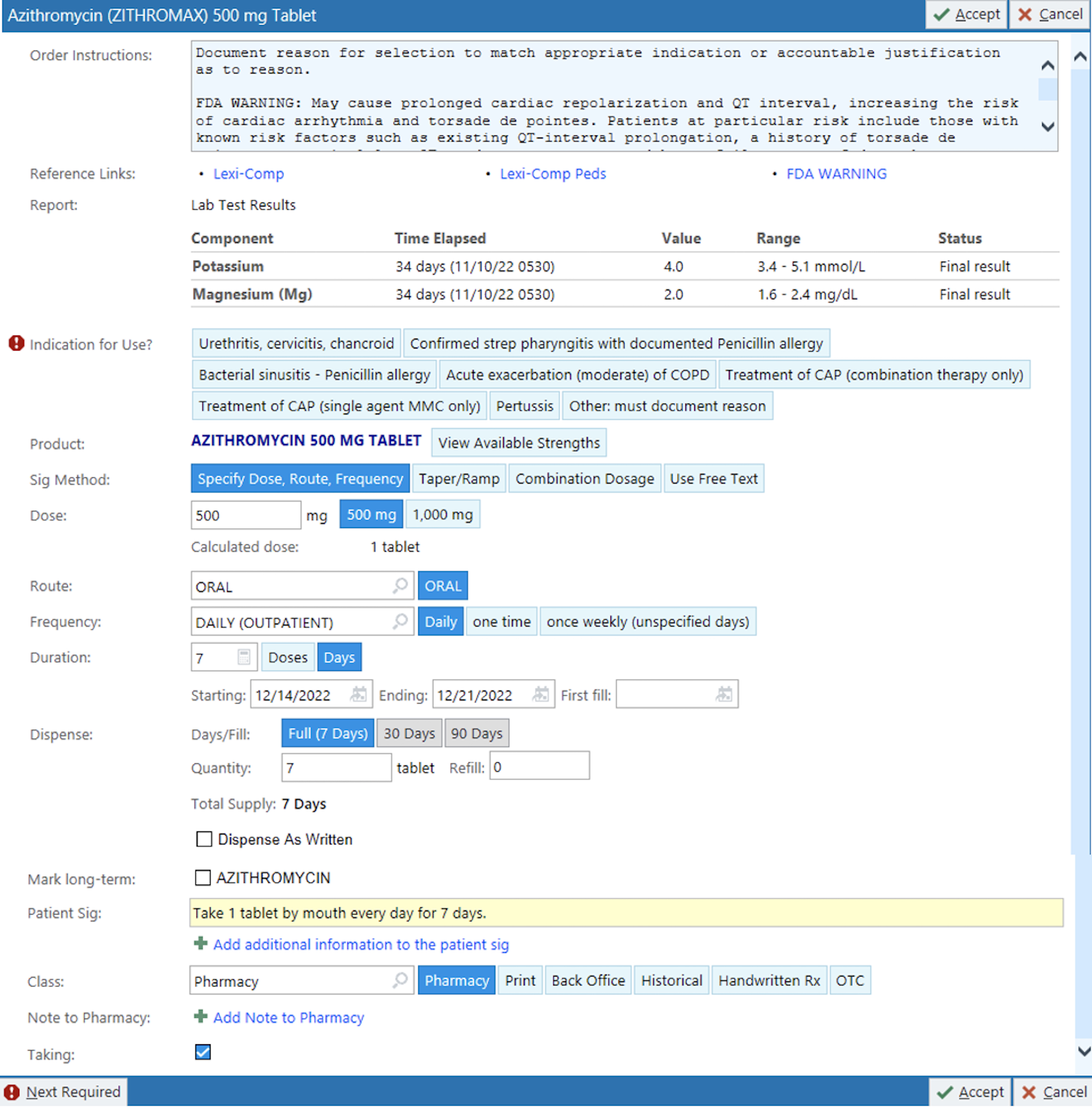

Supplement: Supplementary file 1 [file ashsup.zip › S2732494X23001407sup002.jpg]

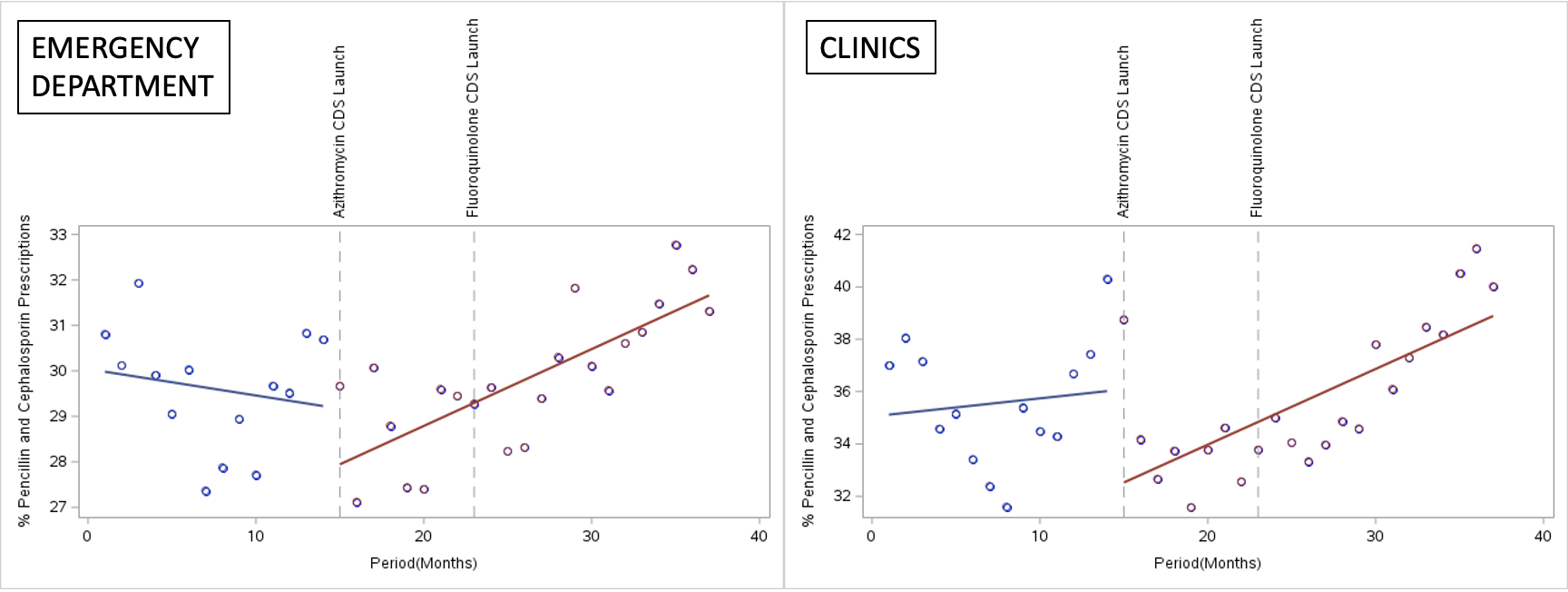

Supplement: Supplementary file 1 [file ashsup.zip › S2732494X23001407sup003.jpg]
